# The Effect of Lidocaine Splash Block Followed by Suspensory Ligament Massage in Female Dogs Undergoing Ovariohysterectomy: A Prospective Study †

**DOI:** 10.3390/ani14233522

**Published:** 2024-12-05

**Authors:** Eugenia Flouraki, Epameinondas Loukopoulos, Dimitrios Gougoulis, Ioannis Savvas, Chrysoula Margeti, Konstantina Karagianni, Vassiliki Tsioli

**Affiliations:** 1 Surgery Clinic, Faculty of Veterinary Medicine, University of Thessaly, 43100 Karditsa, Greece; margeti@uth.gr (C.M.); konkara.vet@gmail.com (K.K.); vtsioli@uth.gr (V.T.); 2 Clinic of Medicine, Faculty of Veterinary Medicine, University of Thessaly, 43100 Karditsa, Greece; dgoug@uth.gr; 3 Companion Animal Clinic, School of Veterinary Medicine, Aristotle University of Thessaloniki, 54627 Thessaloniki, Greece; isavas@vet.auth.gr

**Keywords:** analgesia, dogs, lidocaine, ovariohysterectomy, splash block, suspensory ligament

## Abstract

The administration of lidocaine as part of multimodal analgesia in dogs undergoing ovariohysterectomies demonstrates variable analgesic efficacy. The aim of this prospective, double–blinded clinical study was to evaluate the effectiveness of lidocaine 2% irrigation on the ovarian suspensory ligament, followed by localised massage, in achieving sufficient intraoperative analgesia. Thirty-eight female dogs were randomly divided to two groups and received either a splash block of 0.5 ml lidocaine 2% on each ovarian suspensory ligament (GL) or an equal volume of saline (GNS). All dogs were premedicated with dexmedetomidine (5 μg·kg^−1^) and buprenorphine (20 μg·kg^−1^) intramuscularly and meloxicam (0.2 mg·kg^−1^) subcutaneously. Anaesthesia was induced with propofol and maintained with isoflurane. The surgical procedure was initiated after an adequate depth of anaesthesia was achieved. Heart rate (HR), respiratory rate (*f*_R_) and mean arterial pressure (MAP) were continuously monitored. Fentanyl (2 μg·kg^−1^) was administered as rescue analgesia in case of a 30% increase in HR, *f*_R_, or MAP, compared to their values before manipulation of the ovaries. Data were analysed using a mixed model for repeated measures ANOVA and Pearson’s Chi-Square test. The need for rescue analgesia differed significantly between the two groups (*p* < 0.0005). In GNS, 89.47% (17/19) of the dogs required rescue analgesia, compared to 10.53% (2/19) of the dogs in GL. The relative risk of rescue for the GNS compared to the GL was approximately 8.5. In conclusion, intraoperative analgesia during ovariohysterectomies in dogs can be enhanced using a lidocaine 2% splash block combined with massage of the ovarian suspensory ligament.

## Simple Summary

Ovariohysterectomy is a common elective surgical procedure performed on companion animals. This study aimed to determine whether lidocaine, a topical anaesthetic agent splashed onto the ovarian ligament, followed by a localised massage of the ligament, could provide sufficient analgesia in dogs undergoing ovariohysterectomy. A total of 38 bitches were allocated to two groups. The lidocaine group received 0.5 mL of lidocaine 2% on the suspensory ligaments of the ovaries followed by a one-minute massage, while the placebo group received 0.5 mL of saline followed by a one-minute massage. If signs of pain were observed, rescue analgesia with fentanyl was administered. The outcome of the study revealed that irrigation with lidocaine resulted in fewer painful reactions during ovarian manipulation, reducing the need for rescue analgesia, while anaesthesia was smooth, with no complications. The use of topical irrigation of lidocaine onto the suspensory ligaments of the ovaries is a low-cost, easy-to-perform technique that provides sufficient intraoperative analgesia in dogs undergoing ovariohysterectomies.

## 1. Introduction

The extensive variety of analgesic drugs and techniques available for managing pain in animals poses a challenge for clinicians in choosing the most suitable option for effective pain management. In practice, this choice is influenced by various factors including the type of surgery, the clinician’s past experiences and knowledge of specific drugs or techniques, drug availability, legal legislation and restrictions, associated side effects, and cost. The existing literature supports the use of multimodal analgesia in cases where the effective control of anaesthetic depth is challenging. Multimodal analgesia involves the use of a combination of analgesic drugs targeting different points along the pain pathway [1,2,3,4]. This approach aims to manage pain effectively while minimising the side effects of combined drugs [1,3,4]. The main component of multimodal analgesia is locoregional anaesthesia [1,5]. Its key benefit is the sparing effect on general anaesthetics and the reduced dose requirements of anaesthetic and analgesic drugs [3,6,7,8,9].

Ovariohysterectomy in dogs is a frequent elective surgical procedure in veterinary medicine. It is characterised by its moderate pain induction, primarily stemming from associated tissue damage that triggers painful stimuli [10,11,12,13]. Traction and ligation of the ovarian ligament, regarded as particularly intense noxious stimuli, contribute significantly to pain perception [14,15,16]. In cases where the anaesthetic depth and analgesia prove inadequate, a range of painful signs or excitement manifestations may occur, spanning from increased heart and respiratory rate to pronounced abdominal straining and movement [14,15,16,17,18,19].

Various local anaesthetics, including ropivacaine, bupivacaine, and lidocaine have been used to enhance analgesia in ovariohysterectomies and other procedures. Specifically, lidocaine has been employed in clinical practice during ovariohysterectomies, ovariectomies, and orchiectomies in different animal species, yielding various outcomes [6,20,21,22,23,24,25,26,27,28]. The local administration of lidocaine induces a reversible blockade of impulse transmission along central and peripheral nerve pathways [3,29,30,31,32,33,34]. This administration results in a notable reduction in both intraoperative and postoperative pain [7,8,25,26,33]. The current literature strongly advocates the use of lidocaine, or other local anaesthetics, in most surgical procedures, particularly endorsing their efficacy as adjunctive analgesia in reproductive interventions such as ovariectomies and orchiectomies [6,8,13,21,35,36].

Our study aims to assess the efficacy of lidocaine irrigation on the suspensory ligament of the ovary, followed by localised massage, in achieving sufficient intraoperative analgesia. Specifically, the investigation focuses on whether lidocaine can effectively diminish the need for intraoperative rescue analgesia. Our hypothesis suggests that the irrigation of lidocaine 2% along with massaging of the suspensory ligament would enhance intraoperative analgesia effectiveness.

## 2. Materials and Methods

### 2.1. Animals

This study was approved by the Animals Ethics Committee (EDEXZO, number: 174, date: 15 March 2024) of the Department of Veterinary Medicine of the University of Thessaly (UTH). A total of 38 female dogs of various breeds presented for scheduled elective ovariohysterectomy were included in this prospective, randomised, and double-blinded clinical study. All dogs were client-owned, and all owners provided a written informed consent for inclusion in the study. All dogs included in the study were aged between one and six years and weighed 4 to 35 kg. They were of good health and were listed with an American Society of Anesthesiologists (ASA) physical status of 1. The exclusion criteria were female dogs with underlying medical conditions, dogs that had undergone previous laparotomies, and dogs that had received analgesics or anti-inflammatory drugs within the last 30 days. Additionally, dogs weighing less than 4 kg were excluded from the study. This weight range was carefully chosen to ensure that the lidocaine dosage remained within safe limits, thereby preventing any risk of exceeding the maximum dose. Deep-chested dog breeds, such as Doberman Pinschers and Greyhounds, were excluded from the study due to their unique anatomical characteristics. These characteristics can complicate the handling of the ovaries during ovariohysterectomy, making it challenging to minimise manipulation of both the ovaries and ligaments to prevent nociception prior to lidocaine administration and massage of the ligaments. Brachycephalic breeds, including Bulldogs and Pugs, were also excluded because they exhibit increased vagal tone compared to non-brachycephalic dogs [37]. This heightened vagal tone makes brachycephalic dogs more susceptible to bradycardia during general anaesthesia, and thus they may need lower doses of alpha-2 adrenergic agonists [38]. Additionally, manipulation of the ovaries can further affect these dogs, as the ovaries are innervated by both sympathetic and parasympathetic nerve fibres. Moreover, the administration of fentanyl to anaesthetised brachycephalic dogs can exacerbate the effects of their predominant vagal tone [37]. All dogs included in the study underwent a comprehensive clinical assessment. Age, body weight (BW), rectal temperature (RT), heart rate (HR), respiratory rate (*f*_R_), thoracic auscultation, mucous membrane colour, capillary refill time, and femoral pulse were recorded, and complete blood count, the serum biochemical profile of creatinine (CREAT), blood urea nitrogen (BUN), alanine transaminase (ALT), alkaline phosphatase (ALP), blood glucose (GLU), albumin (ALB), and total protein (TP) were measured the day before the experiment. If abnormal findings were detected, the dog was excluded from the study. Food and water were withheld for 8 to 10 h and 2 h, respectively, before premedication. Every dog was admitted to the hospital at least 1 h prior to the study and remained on the premises with its owner in a quiet examination room.

### 2.2. Anaesthesia and Surgical Procedure

All dogs included in the study were randomly allocated to two groups, each consisting of 19 dogs. Randomisation was performed using an Excel spreadsheet (Microsoft Office 2019 Professional Plus, Version 2410), which generated a random allocation sequence. This sequence was used to assign the dogs to the treatment groups to ensure unbiased distribution. Dogs assigned to the lidocaine group (GL) received topical irrigation (splash block) with 0.5 mL of lidocaine 2% (Xylozan 2%, Demo S.A., Kryoneri, Greece) on each suspensory ligament. Dogs assigned to the control group (GNS) received irrigation of each suspensory ligament with an equivalent volume of saline (Sodium Chloride 0.9%). The study was conducted in a double-blind manner, with both the anaesthesiologist and surgeon unaware of the treatment administered to each dog. To maintain procedural integrity and measurement objectivity, lidocaine or saline was covertly prepared before the experiment by a third examiner who was not involved in decision making throughout the procedure.

All dogs were premedicated with a combination of dexmedetomidine (Dexdomitor, Elanco, Chalandri, Greece) at a dose of 5 μg·kg^−1^ and buprenorphine (Bupaq, Neocell, Metamorfosi, Greece) at a dose of 20 μg·kg^−1^. The drugs were mixed in the same syringe and administered intramuscularly (IM) into the semimembranosus muscle. The animals were kept under observation in the same room for 20 min. Subsequently, the left cephalic vein was catheterised using an aseptic technique with an appropriately sized intravenous catheter, and Lactated Ringer’s solution was infused at 5 mL·kg^−1^·h^−1^ throughout the procedure or adjusted as needed based on intraoperative requirements. Anaesthesia was induced with propofol (Propofol 1% MCT/LCT, Fresenius Kabi, Marousi, Greece) at a dose of 1 mg·kg^−1^ intravenously (IV), followed by incremental doses of 1 mg·kg^−1^ to allow endotracheal intubation. Subsequently, tracheal intubation was facilitated using an appropriately sized cuffed endotracheal tube. The total dose of propofol administered during the induction was recorded. After the induction of anaesthesia, meloxicam (Metacam, Boehringer Ingelheim, Ingelheim am Rhein, Germany) was administered subcutaneously (SC) at a dose of 0.2 mg·kg^−1^. Anaesthesia was maintained with isoflurane in oxygen through a circled anaesthetic circuit, and the vaporiser was set at 1.5% and was adjusted up to 2%. For dogs weighing from 4 to 7 kg, a Bain anaesthetic system was used. All dogs were breathing spontaneously throughout the procedure. Continuous monitoring (Datex Ohmeda S5 Compact patient monitor, GE Healthcare, Helsinki, Finland) was instituted after intubation and included HR, *f*_R_, mean arterial pressure (MAP), haemoglobin oxygen saturation (SpO_2_), end-tidal carbon dioxide (PE′CO_2_), and inspired and expired isoflurane fractions (FIIso and FE′Iso). Noninvasive arterial blood pressure was measured using the oscillometric method, with an appropriate cuff placed above the carpus. The HR, *f*_R_, MAP, PE′CO_2_, FIIso, and FE′Iso were recorded at one-minute intervals. The assessment of anaesthetic depth involved an evaluation of the palpebral reflex, the absence of jaw tone, and eye position. Animals were positioned in dorsal recumbency and left without handling, with the isoflurane vaporiser gradually adjusted from 1.5% and up to 2%, as needed. The surgical procedure was initiated when the FE′Iso reached and remained stable at 1.2% for 10 min. Throughout the anaesthetic period, the isoflurane vaporiser was adjusted when needed from 1.5% to 2% to maintain the FE′Iso at 1.2%; however, it never exceeded 2%.

The same surgeon performed all procedures. Ovariohysterectomy was performed through a midline laparotomy approach. Subsequently, the left ovary was identified without traction manipulations, and the suspensory ligament was irrigated with 0.5 mL of either lidocaine or saline, followed by a gentle one-minute massage and an additional 1 min of waiting time. At the end of the entire two-minute duration, including both massage and waiting periods, ligation and dissection of the ovarian pedicle were performed following the standard procedural protocol. In the event of a 30% intraoperative increase in at least one of the following vital haemodynamic parameters—HR, *f*_R_, or MAP—relative to their previous values before ovarian manipulation, an IV bolus of fentanyl (Fentanyl, Janssen-Cilag, Pefki, Greece) at a dose of 2 μg·kg^−1^ was administered as rescue analgesia. In case of unsuccessful rescue analgesia and an inadequate depth of anaesthesia, the dog was excluded from the study and replaced by the next dog in order. Less than 30% of the changes in the parameters during surgical stimulation were ignored. The same procedure was performed for the right ovary. Subsequently, the uterus was ligated and removed, followed by closure of the abdominal cavity and skin in a routine manner. After the surgical operation was completed, anaesthesia was discontinued, and the dog was left to recover. Extubation was performed upon coughing and swallowing, in response to the presence of the endotracheal tube.

Time points for statistical analysis were defined as T1, initiation of anaesthesia maintenance with isoflurane; T2, attainment of a 1.2% FE′Iso; T3, 10 min after a stable FE′Iso at 1.2% and beginning of the surgical procedure; T4, irrigation of the suspensory ligament of the left ovary; T5, surgical manipulation of the left ovary; T6, irrigation of the suspensory ligament of the right ovary; T7, surgical manipulation of the right ovary; T8, completion of surgical procedure and discontinuation of anaesthesia (Table 1). In addition, the duration of the surgical procedure, and the duration of anaesthesia, were documented.

All dogs were discharged the same day. For postoperative pain management, all animals were prescribed meloxicam at a dose of 0.1 mg·kg^−^^1^ once daily for four days and tramadol at a dose of 2 mg·kg^−^^1^ twice daily for two days.

### 2.3. Statistical Analysis

The determination of the requisite sample size was conducted using computer software (G*power 3.1.9.4, Universität Kiel, Kiel, Germany). The nature of the comparison involves categorical data, wherein each canine subject was categorised based on the presence or absence of a reaction to painful stimuli. To ensure robust statistical analysis, we conducted a pilot study to estimate the effect size, which yielded a value of 0.6. Consequently, the Chi-Square goodness-of-fit test for categorical data was employed to determine the requisite sample size. For a statistical power of 95% at a significance level (alpha) of 0.05, the calculated minimum sample size for this study was 38 animals, with 19 subjects allocated to each group (*n* = 19). This determination ensured the robustness of the study to detect meaningful differences between the proportions of dogs exhibiting reactions to painful stimuli in the specified groups.

A Chi-Square test for independence was conducted to examine the association between groups (GNS vs. GL) and the occurrence of rescue. Additionally, the relative risk (RR) was calculated to quantify the increased risk of rescue for GNS compared to GL.

Data analysis was performed using IBM SPSS Statistics for Windows, version 29 (IBM Corp., Armonk, NY, USA).

The normality of physiological parameters was assessed using the Shapiro–Wilk test for each time point within each group. When the data did not meet the assumption of normality, proper transformations were applied to normalise the distributions. Specifically, to achieve normality for the HR and *f*_R_ data: a logarithmic transformation was applied for the HR data, and a square root transformation was applied for the *f*_R_ data.

A repeated-measures ANOVA was conducted to examine the effects of time and treatment group on physiological parameters. This analysis was appropriate due to the repeated measurements of HR, *f*_R_, and MAP at eight different time points (T1, T2, T3, T4, T5, T6, T7, T8) for each participant. The independent variable was the treatment group (treated vs. control), and the within-subjects, independent variable factor was time. To account for potential violations of the sphericity assumption, Mauchly’s Test of Sphericity was performed, which indicated a violation (*p* < 0.001). Consequently, the Greenhouse–Geisser correction was applied to adjust the degrees of freedom for the within-subject tests. The analysis included multivariate tests to evaluate the main effect of time, the main effect of group, and the interaction between time and group. The statistical significance was set at α = 0.05. The results were interpreted based on the corrected degrees of freedom and *p*-values.

## 3. Results

In this study, a total of 38 dogs were used. All enrolled dogs successfully completed the study, except for two dogs in the GNS that required an additional dose of propofol (1 mg·kg^−1^), apart from the administration of rescue analgesia with fentanyl (2 mg·kg^−1^), during manipulation of the first ovary. These interventions excluded the animals from the preset anaesthetic protocol, so they were excluded from the study and replaced by the next in order. No significant side effects were observed in relation to the surgical procedure, premedicants, or lidocaine administration, and the ovaries were removed without complications. All dogs recovered uneventfully, with no postoperative complications associated with the anaesthetic protocol or lidocaine administration. Owners were given instructions for telephone contact in the event of observing any adverse effects regarding their dogs, and a follow-up appointment was scheduled for reassessment after one week. Re-examination one week after the initial procedure revealed no abnormalities in any of the enrolled animals. No complaints or changes in animal behaviour were reported by the owners.

The mean age (±standard deviation SD) of the study population was 2.73 (±1.41) years and the mean body weight was 16.05 (±9.83) kg. The mean value for the total dose of propofol (mg) used for induction was 43.15 (±25.13), while the mean dose of propofol per kilogram of body weight (mg·kg^−1^) was 2.94 (±1.03). The mean duration of anaesthesia was 45.89 (±3.68) min and the mean duration of surgery was 29.81 (±3.60) min. Detailed descriptive results for each group are comprehensively presented in Table 2.

**Figure 1:**
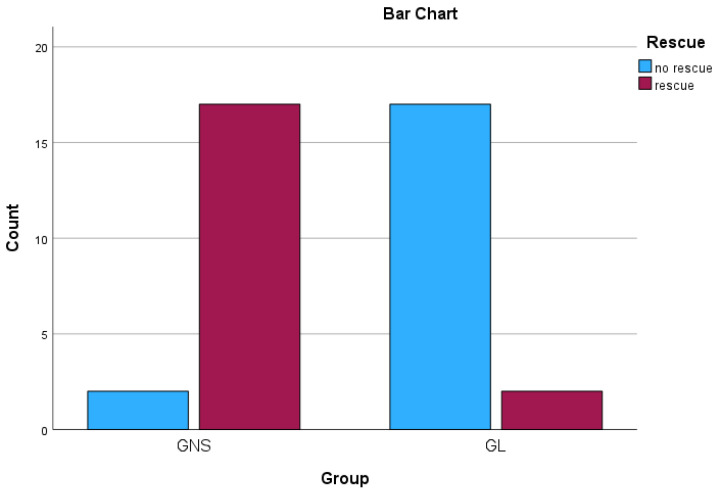
The proportions of 38 dogs undergoing ovariohysterectomy that required rescue analgesia by group (*n* = 19), after receiving a splash block on the suspensory ligaments with 0.5 mL of either lidocaine 2% (GL) or saline (GNS), followed by a gentle one-minute massage.

Among the 38 dogs included in this study, 19 (50%) exhibited an intraoperative increase of 30% or more in vital hemodynamic parameters (HR, *f*_R_ and MAP) immediately after the surgical manipulation of the ovaries, relative to their previous values prior to ovarian manipulation. The need for rescue analgesia differed significantly between the two groups (*p* < 0.0005). A total of 17/19 (89.47%) dogs in the GNS required rescue analgesia intraoperatively, compared to 2/19 (10.53%) in the GL (Figure 1). The relative risk (RR) of rescue for the GNS compared to the GL was calculated to be approximately 8.5. This indicates that the GNS had a significantly higher risk of requiring rescue analgesia, being 8.5 times more likely to receive rescue analgesia, than the GL. Furthermore, 10/17 (58.82%) of the dogs that required rescue analgesia in the GNS ended up receiving a bolus of fentanyl (rescue), immediately after the surgical manipulation of each ovary, while the remaining 7/17 (41.18%) of them required rescue analgesia only after the surgical manipulation of the first ovary. In the GL, one out of the two dogs that required rescue analgesia received two doses of fentanyl, each after the surgical manipulation of the respective ovary, while the other one received only one dose of fentanyl immediately after the surgical manipulation of the first ovary.

The descriptive results regarding the mean HR of the study population according to group are presented in Table S1 of the Supplementary Materials.

**Figure 2:**
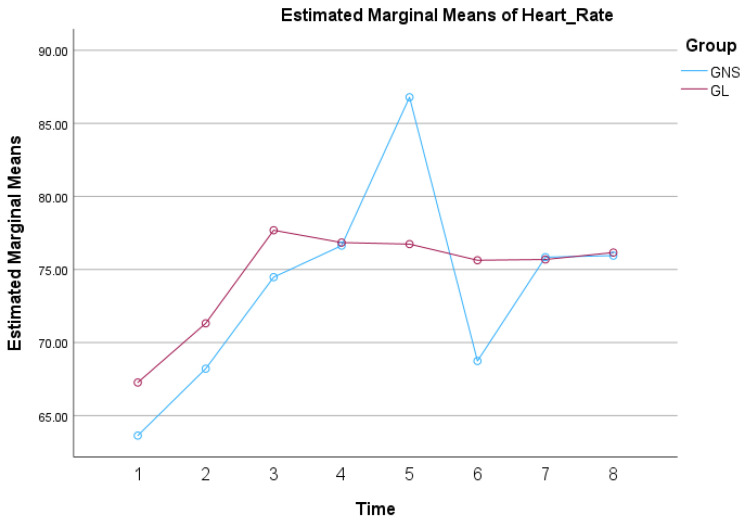
Mean HR values by group over time in 38 dogs undergoing ovariohysterectomy that received a splash block on the suspensory ligaments with 0.5 mL of either lidocaine 2% (GL) or saline (GNS), followed by a gentle one-minute massage.

The HR within each group significantly changed over time during surgery (*p* < 0.001), and this change differed between the two groups (*p* = 0.015). However, the HR values overall were not significantly different between the two groups (*p* = 0.850). These results indicate that both groups experienced changes in HR during surgery and the pattern of these changes over time varied between the groups. Moreover, in GNS, the mean values for HR were significantly higher at T5 compared to all the other time points (*p* < 0.001) and at T7 compared to T6 (*p* = 0.002). In GL, there were no significant differences in HR, among all time points. The profile plots of the estimated marginal of mean HR over time for both groups, showing the overall trend and interaction effects, are presented in Figure 2.

The descriptive results regarding the mean *f*_R_ of the study population according to group are presented in Table S2 of the Supplementary Materials.

**Figure 3:**
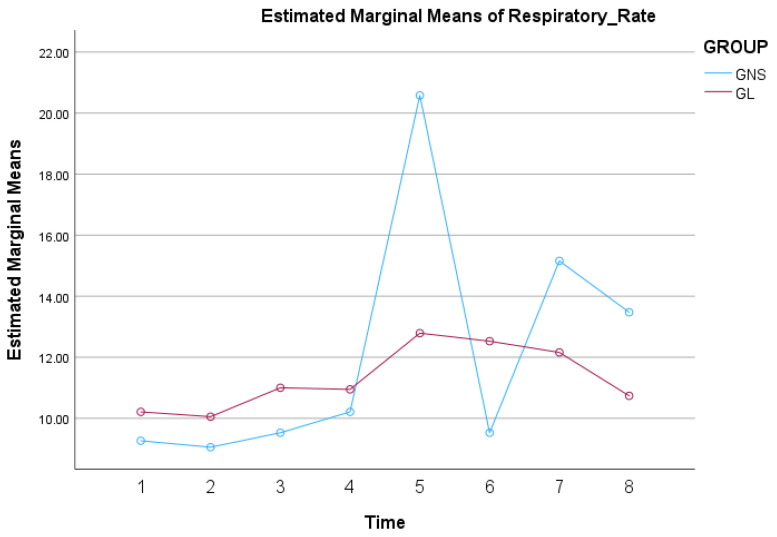
Mean *f*_R_ values by group over time in 38 dogs undergoing ovariohysterectomy that received a splash block on the suspensory ligaments with 0.5 mL of either lidocaine 2% (GL) or saline (GNS), followed by a gentle one-minute massage.

The analysis revealed significant variations in mean *f*_R_ within each group over time (*p* < 0.001). However, there were no significant differences between the two groups overall (*p* = 0.807). These findings suggest that time-related factors significantly impacted respiratory rates and that this impact varied between the two groups, though not significantly. In contrast, a significant difference was detected between groups at T5 (*p* = 0.008). Furthermore, in GNS the mean *f*_R_ was significantly higher at T5 compared to all the other time points (*p* < 0.001) and at T7 compared to T6 (*p* = 0.001). In GL, there were no significant differences in mean *f*_R_, among all time points. The profile plots of the estimated marginal mean *f*_R_ values over time for both groups, showing the overall trend and interaction effects, are presented in Figure 3.

The descriptive results regarding the mean MAP of the study population according to group are presented in Table S3 of the Supplementary Materials.

**Figure 4:**
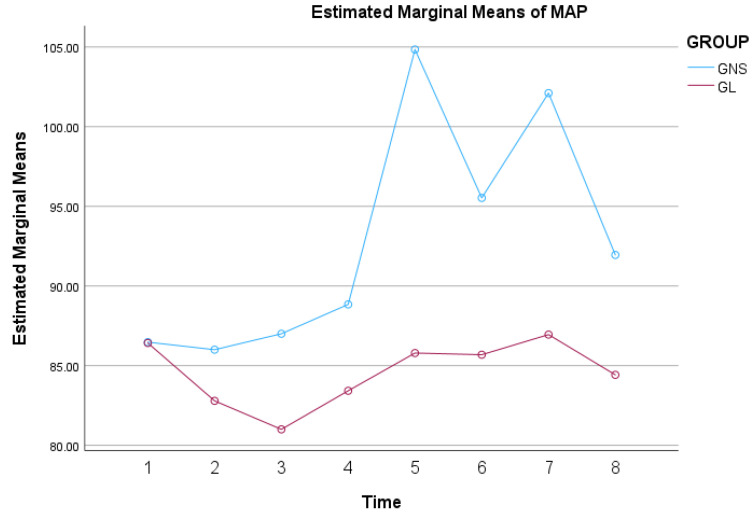
Mean MAP values by group over time in 38 dogs undergoing ovariohysterectomy that received a splash block on the suspensory ligaments with 0.5 mL of either lidocaine 2% (GL) or saline (GNS), followed by a gentle one-minute massage.

MAP significantly changed over time within each group during surgery (*p* < 0.001), and this change differed between the two groups (*p* = 0.002). Additionally, there was a significant main effect of group on MAP, indicating that overall MAP levels were different between GNS and GL (*p* = 0.002). These results indicate that both groups experienced significant changes in MAP during surgery, and the pattern of these changes and the overall MAP levels differed between the two groups. More specifically, mean MAP revealed a significant difference between groups at T5 (*p* < 0.001), T6 (*p* = 0.003), T7 (*p* < 0.001), and T8 (*p* = 0.007). Furthermore, in group GNS, the mean MAP was significantly higher at T5 compared to all the other time points (*p* < 0.001), and at T7 compared to T6 (*p* < 0.001). On the other hand, in group GL, there were no significant differences detected concerning mean MAP when T5 compared to T4 (*p* = 0.252) and T7 compared to T6 (*p* = 0.643). The profile plots of the estimated marginal mean MAP values over time for both groups, showing the overall trend and interaction effects, are presented in Figure 4.

## 4. Discussion

Ovariohysterectomy is a commonly performed elective surgical procedure in veterinary medicine, characterised by moderate pain due to tissue damage and nociceptive stimuli [10,11,12,13]. The use of local anaesthetics, such as lidocaine, as an adjunctive analgesic in animals undergoing surgeries of the reproductive system is a technique with a low incidence of complications and low cost [29,39,40]. However, its efficiency in ovariohysterectomies and ovariectomies remains a subject of controversy. Lidocaine has been variably applied in ovariectomies and ovariohysterectomies, including the infusion or topical irrigation of the mesovarium and the ovary [20,21,23,41], an intraperitoneal “splash block” [6,40,42], or as incisional administration [6,40,42]. In our study, the local irrigation of lidocaine 2% on the suspensory ligament of each ovary significantly reduced the need for rescue analgesia when compared to the control group. In the lidocaine group, 17/19 dogs exhibited less than a 30% change in their vital parameters such HR, *f_R_*, and MAP, during the manipulation and ligation of the ovaries. These results are in line with the study by Cicirelli et al. (2022), where a splash block of lidocaine onto the mesovarium resulted in reduced need for rescue systematic analgesia compared to the placebo group in dogs undergoing laparoscopic ovariectomies [21]. Moreover, a study comparing lidocaine local irrigation and infusion into the mesovarium found that lidocaine enhanced surgical analgesia with both methods, and although there was no control group to compare the outcomes, the need of rescue analgesia was still very low [41]. On the contrary, both studies by Bubalo et al. (2008) and Gomes et al. (2024) observed that lidocaine infiltration into the mesovarium did not yield additional analgesic benefits compared to animals administered saline, even though a larger volume of lidocaine was infiltrated in the study by Gomes et al. [20,23]. Bubalo et al. (2008) rationalised the outcome as a result of the synergic effect of the analgesic protocol used in the study [20]. Specifically, methadone, a strong μ-receptor full agonist, administered in premedication combined with an incisional infiltration of lidocaine, may have masked the additional analgesic effect of lidocaine infiltration. A similar rationalisation was provided by Gomes et al. (2024), where morphine was used instead of methadone [23]. However, in the study by Cicirelli et al. (2022), methadone was implemented in premedication and an additional bolus and constant-rate infusion of fentanyl was added in the anaesthetic protocol, and yet, they succeeded in finding an analgesic effect of lidocaine infusion into the mesovarium [21]. In our study, a partial μ-agonist opioid, buprenorphine, was administered in premedication. Buprenorphine has been used as part of premedication protocols in dogs undergoing ovariohysterectomies, and the intraoperative monitoring was unremarkable [43,44]. The existing literature supports the efficacy of anaesthetic protocols using buprenorphine for canine ovariohysterectomy, demonstrating favourable outcomes. Several studies by Shih et al. (2008), Nunamaker et al. (2014), and Slingsby et al. (2011) provide evidence of successful analgesic management in ovariohysterectomies and ovariectomies in dogs [9,43,44]. In our study, the use of buprenorphine in combination with the irrigation of lidocaine on the suspensory ovarian ligaments reduced the need for intraoperative rescue analgesia.

We also included meloxicam as part of the preoperative analgesic protocol. Current practices for most elective surgeries, including canine ovariohysterectomies, recommend administering a non-steroidal anti-inflammatory drug (NSAID) such as meloxicam perioperatively [45]. Ideally, NSAIDs are given preoperatively to enhance the benefits of pre-emptive analgesia [46]. Additionally, combining an NSAID with an opioid is known to support multimodal analgesia [47]. Furthermore, a study by Nunamaker et al. (2014) indicated that the combination of buprenorphine and meloxicam resulted in only one out of twenty dogs (5%) requiring rescue analgesia, a notably lower rate compared to other protocols [43]. It has also been suggested that meloxicam alone can provide adequate postoperative analgesia in canine ovariohysterectomies [48].

The use of opioids in veterinary medicine has been included in almost every anaesthetic protocol as part of a balanced multimodal analgesia [49]. However, over the last few years, the existing literature has indicated various concerns regarding opioid use, both in human and veterinary medicine. Most importantly, opioids are associated with numerous adverse effects, most of which are primarily drug- and dose-dependent. Nausea, gastroesophageal reflux, vomiting, bradycardia, and respiratory depression, accompanied with hypercapnia and even alterations in immune system functioning, which can negatively affect infectious or cancerous pathologies, are some of the most significant adverse effects regarding opioid use [5,50,51,52,53,54,55,56]. In human medicine, both the preoperative and intraoperative use of opioids as means of analgesia have been associated with an increase in postoperative pain and longer hospitalisation times [57,58,59,60,61]. In addition, periods of opioid shortages (opioid crises) as well as many countries’ restriction regulations, regarding the use of opioids in veterinary medicine, have created many problems in treating animal pain, especially in the perioperative period. To compensate for these issues, both human and veterinary medicine have shifted towards opioid-free or opioid-sparing anaesthesia, where opioid use is excluded or minimised while using other means of analgesia, such as NSAIDs and local anaesthetics, or other adjunctive analgesics. Several studies in veterinary medicine have demonstrated the analgesic potency of many opioid-free and opioid-sparing anaesthesia protocols in a variety of surgical procedures [8,21,40,41,42,62,63,64,65]. The use of buprenorphine, an opioid with fewer legal restrictions in most countries, in combination with of a local irrigation of lidocaine on the ovarian suspensory ligaments and the addition of an NSAID, may prove efficient intraoperatively for dogs undergoing ovariohysterectomies, as was demonstrated in our study.

In our study, we used a partial μ-agonist for premedication and a μ-full agonist for rescue analgesia intraoperatively. We recognise the pharmacological concerns regarding the use of buprenorphine concurrently with full μ-agonists such as fentanyl for rescue analgesia, due to potential competitive interactions at μ-opioid receptors [66]. However, while this concern has been raised, the current evidence does not fully support a significant antagonistic interaction at clinically relevant doses. Studies in both human and veterinary medicine have shown that at therapeutic doses, combining buprenorphine with full μ-agonists like fentanyl, morphine, methadone, and hydromorphone generally produces additive and sometimes synergistic analgesic effects [67,68,69,70]. Antagonistic effects tend to occur only when buprenorphine is used at doses exceeding the analgesic range, indicating that interactions at typical clinical doses are generally supportive rather than inhibitory [69,71]. For example, Kögel et al. (2005) found that buprenorphine’s partial agonistic properties only exhibited antagonistic effects with full μ-agonists when high doses exceeding therapeutic ranges were combined [68]. Furthermore, studies in both human and veterinary medicine demonstrate that it is feasible to transition from buprenorphine to full μ-agonists without a significant refractory period or loss in analgesic efficacy [68,72]. Additionally, pharmacodynamic studies suggest that buprenorphine’s binding at μ-receptors allows it to coexist with other opioids, as the competitive displacement of buprenorphine occurs at high but clinically feasible opioid concentrations [73]. In a study in dogs, Hunt et al. (2013) used methadone for post-operative rescue analgesia in dogs undergoing orthopaedic surgery, with buprenorphine administered in the premedication and postoperative periods, and suggested that one dose of rescue methadone IM was sufficient enough to produce analgesia, while the postoperative analgesic protocol with buprenorphine was not discontinued [67]. In our study, the co-administration of buprenorphine and fentanyl as rescue analgesia resulted in a maximum of two doses of fentanyl, one for each ovarian manipulation. Only two dogs required an extra dose of propofol to adjust the anaesthetic depth.

The safe margins of lidocaine dosage in dogs are wide and range from 1 to 10 mg·kg^−1^ [1,6,29,40]. In our study, we used 0.5 mL lidocaine 2% for each ovary regardless of the size of the dogs included in the study. This corresponds to the smallest dogs weighing 4 kg receiving a dose of 5 mg·kg^−1^ lidocaine in total, which is within a safe dose margin. The safety and pharmacokinetics of lidocaine used in an intraperitoneal and incisional manner have been studied by Wilson et al. (2004) [40]. The study demonstrated that a dose of 8.8 mg·kg^−1^ splashed intraperitoneally and 2 mg·kg^−1^ splashed on the incision wound resulted in no adverse consequences or toxicity in dogs undergoing ovariohysterectomies. The choice not to calculate the dose of lidocaine in relation to the body weight of the dogs included in our study might have resulted in failure to prove an effective analgesic result. However, no correlation was found between body weight and treatment. It appears that the massage performed in the suspensory ligaments and the time given for the drug to act was efficient. Similarly, several studies have proposed the use of a standard volume of lidocaine or other local anaesthetics in dogs undergoing ovariohysterectomies; however, the outcomes vary [20,23].

The onset time of lidocaine has been reported to range from 2 to 5 min [74]. In our study the total waiting time before the surgical manipulation of the ovaries was 2 min. A similar time has been reported in two studies, where lidocaine was either instilled or infused into the mesovarium and the ceasing of all surgical manipulation lasted 90 s. On the contrary, Bubalo et al. (2008) and Gomes et al. (2024) reported a 5 min waiting time before surgical manipulations, after the infusion of lidocaine into the mesovarium. However, no additional analgesic effect resulting from the administration of lidocaine was demonstrated [20,23].

Lidocaine has also been used as a “splash block” intraperitoneally in dogs undergoing ovariohysterectomies. Carpenter et al. (2004) found no differences in intraoperative and postoperative pain following intraperitoneal lidocaine administration [6], while Brioschi et al. (2023) and Wilson et al. (2004), reported that this technique effectively succeeds in reducing postoperative pain without any adverse effects or signs of toxicity [40,42]. Several other local anaesthetics, such as bupivacaine and ropivacaine, have been used intraperitoneally to manage postoperative pain in dogs undergoing ovariohysterectomies [6,42,75,76,77,78]. In most of the studies performed in dogs undergoing ovariohysterectomies, the local anaesthetic was infused in the peritoneal cavity prior to the closure of the linea alba, and all measurements were related to the assessments of postoperative pain [6,42,76,77,78]. In a study by Campagnol et al. (2012), bupivacaine was dripped onto the ovaries before surgical manipulations; however, no information is given for the need for intraoperative rescue analgesia [75]. Nonetheless, the instillation of bupivacaine resulted in an improved analgesic outcome postoperatively, and in a trend of less required rescue analgesia postoperatively.

Various locoregional techniques have also been implemented in veterinary medicine to improve pain management in dogs undergoing ovariohysterectomies. Numerous techniques have been tested in the search for new multimodal anaesthesia approaches to manage intraoperative and immediate postoperative pain in ovariohysterectomies in dogs and other major abdominal surgeries. The quadratus lumborum (QL) block is an ultrasound-guided technique, consisting of a blockade of the intercostal nerves of the abdominal wall, after local anaesthetic injection near the QL muscle, providing both somatic and visceral abdominal analgesia [65]. This technique has been used in ovariohysterectomies in dogs, with promising results regarding intraoperative pain control and the likelihood of rescue analgesia administration [65,79]. The transversus abdominis plane (TAP) block is another technique used in abdominal surgeries, with existing data confirming its analgesic contribution in veterinary medicine as well [80,81,82]. Furthermore, epidural anaesthesia is a proven method for achieving sensory blockade in large areas on the abdominal wall, and is already being used in ovariohysterectomies in dogs [83,84]. However, the QL block and the TAP block, as well as epidural anaesthesia, are technically challenging procedures in clinical practice, requiring expertise, experience, and specialised equipment for their execution, while adverse effects derived from mistakes in procedures are also significant factors.

Studies in human medicine support the idea that massage can significantly improve the absorption and efficacy of locally applied therapies [85]. The act of massaging can modify the skin’s barrier layer, especially the stratum corneum, to facilitate a faster permeation rate and enhance the amount of drug retained within the tissue [86,87,88]. Furthermore, massage has been shown to increase local blood flow and elevate skin temperature, which in turn promotes drug absorption by increasing molecular movement and altering skin structures to allow deeper penetration [85]. For instance, Hinds et al. (2004) observed that while deep massage did not significantly increase femoral artery blood flow, it elevated blood flow and temperature in the skin, factors that are known to support the absorption of topical therapies [89]. Massage can also contribute to a more extensive spread of the local anaesthetic within the tissue. Studies have also shown that post-injection massage increases the distribution of local anaesthetics during nerve blocks [90]. This may potentially allow the lidocaine irrigated to the suspensory ligament to disperse more effectively through the tissue, enhancing its analgesic effect intraoperatively. Therefore, massaging the suspensory ligament following lidocaine administration may contribute to both an improvement in the absorption and an enhancement of the local effect of the lidocaine. However, this is only a theory extracted from the effects of the massage in the skin layer, and no scientific evidence can be obtained for the effect of the massage on the suspensory ligaments.

Ovarian innervation derives from the intermesenteric and caudal mesenteric plexus for the sympathetic fibres and from the vagus nerve regarding the parasympathetic fibres [74]. In addition, the ovarian plexus contains sensory fibres that transmit nociceptive stimulation from the ovaries to the central nervous system. The ovaries are highly sensitive to pressure due to the established nerve supply [91]. Aβ fibres activated by pressure or light touch are responsible for the transmission of non-nociceptive stimuli in the dorsal horn at laminae III and IV. The activation of these mechanoreceptor fibres can result in the further activation of inhibitory interneurons in the substantia gelatinosa, participating in the gate theory [92,93]. The closing of the “gate” that transmits pain further in the brain can be accomplished either by the activation of the Aβ fibres or by the descending inhibitor pathway. The massage of the suspensory ligaments performed in our study may have activated these mechanoreceptor fibres, enhancing the blockade produced by lidocaine. However, in GNS, the “analgesic” effect of massage without the use of lidocaine was insufficient. Although no specific explanation can be provided on how the massage can enhance the effect of lidocaine, we believe that the activation of the inhibitory nociceptive pathway by the activation of the Aβ fibres in conjunction with the blockade effect of lidocaine may have contributed to the observed outcome. However, further studies are required to explain the possible “analgesic” contribution of the light massage on the suspensory ligaments of the ovaries.

A limitation of our study is that the immediate postoperative period was not assessed for pain; thus, no information can be concluded about the postoperative effect of lidocaine administration on the suspensory ligaments. However, recovery was unremarkable, and upon the re-examination of the dogs a week later, no complaint from the owners was stated regarding changes in the animals’ behaviour. Another limitation of the study is the exclusion of deep-chested and brachycephalic dogs, as well as dogs younger than one year old and older than six, and dogs weighing outside the range of 4–35 kg. The inclusion of these individuals and breeds may have altered the outcome.

## 5. Conclusions

The irrigation of 0.5 mL lidocaine 2% on each ovarian suspensory ligament, followed by localised massage, prior to ovarian manipulation, significantly reduced the occurrence of reactions to painful stimuli during the ligations of the ovaries and minimised the requirements for rescue analgesia [94]. This simple and affordable technique can be easily implemented in daily clinical practice in dogs undergoing ovariohysterectomies, as it requires no specific training or expertise and no specialised equipment. Moreover, it can be beneficial in situations where restrictions, legislation, or even drug shortages make pain management in ovariohysterectomies challenging.

## Figures and Tables

**Table 1:** The specific time points utilised as reference markers for describing the anaesthesia protocol and surgical procedure in the study, involving 38 dogs undergoing ovariohysterectomy that received a splash block on the suspensory ligaments with 0.5 mL of either lidocaine 2% (GL) or saline (GNS) followed by a gentle one-minute massage.

Time Points	Procedure Status
T1	Initiation of anaesthesia maintenance with isoflurane
T2	Attainment of a 1.2% FE′Iso
T3	Commencement of the surgical procedure, subsequent to maintaining FE′Iso at 1.2% for a duration of 10 min
T4	Irrigation of the suspensory ligament of the left ovary
T5	Surgical manipulation of the left ovary
T6	Irrigation of the suspensory ligament of the right ovary
T7	Surgical manipulation of the right ovary
T8	Completion of the surgical procedure and discontinuation of anaesthesia

**Table 2:** Descriptive results (mean ± SD) for age, BW, total propofol dose, propofol dose per kg of BW, duration of anaesthesia, and duration of surgery in 38 dogs undergoing ovariohysterectomy receiving a splash block on the suspensory ligaments with 0.5 mL of either lidocaine 2% (GL) or saline (GNS) followed by a gentle one-minute massage. (*n* = 19).

Variable	Groups		Study Population
	GNS	GL	
Age (years)	2.89 (±1.67)	2.57 (±1.13)	2.73 (±1.41)
Weight (kg)	16.07 (±9.70)	16.04 (±10.23)	16.05 (±9.83)
Total propofol dose (mg)	40.26 (±22.39)	40.05 (±27.91)	43.15 (±25.13)
Propofol dose per kilogram (mg·kg^−1^)	2.79 (±1.19)	3.10 (±0.85)	2.94 (±1.03)
Duration of Anaesthesia (min)	45.15 (±3.76)	46.63 (±3.56)	45.89 (±3.68)
Duration of Surgery (min)	29.21 (±4.13)	30.42 (±2.96)	29.81 (±3.60)

## Data Availability

Data available upon request.

## Supplementary Materials

The following supporting information can be downloaded at: https://www.mdpi.com/article/10.3390/ani14233522/s1, Table S1. Descriptive results (mean ± SD) for HR measurements in 38 dogs undergoing ovariohysterectomy that received a splash block on the suspensory ligaments with 0.5 mL of either lidocaine 2% (GL) or saline (GNS), followed by a gentle one-minute massage. (*n* = 19). Table S2. Descriptive results (mean ± SD) for *f*_R_ measurements in 38 dogs undergoing ovariohysterectomy that received a splash block on the suspensory ligaments with 0.5 mL of either lidocaine 2% (GNS) or saline (GL), followed by a gentle one-minute massage. (*n* = 19). Table S3. Descriptive results (mean ± SD) for MAP measurements in 38 dogs undergoing ovariohysterectomy that received splash block on the suspensory ligaments with 0.5 mL of either lidocaine 2% (GL) or saline (GNS), followed by a gentle one-minute massage. (*n* = 19).
