# Supplementary material for: The Effect of Lidocaine Splash Block Followed by Suspensory Ligament Massage in Female Dogs Undergoing Ovariohysterectomy: A Prospective Study
Source: Animals (Basel). 2024 Dec 5;14(23):3522. doi: 10.3390/ani14233522 (PMC11640144; doi:10.3390/ani14233522)
Supplement: Supplementary file 1 [file animals-14-03522-s001.zip › animals-3252633-supplementary.pdf]

Supplementary materials

Table S1. Descriptive results (mean ± SD) for HR measurements in 38 dogs undergoing ovariohysterectomy that received a splash block on the suspensory ligaments with 0.5 ml of either lidocaine 2% (GL) or saline (GNS), followed by a gentle one-minute massage. (*n* = 19).

Time Point	Group	Mean	Std. Deviation	p-value
T1	GNS	64	17	0.475
	GL	67	14	
	Total	65	15	
T2	GNS	68	17	0.546
	GL	71	14	
	Total	70	16	
T3	GNS	74	16	0.541
	GL	78	16	
	Total	76	16	
T4	GNS	77	16	0.966
	GL	77	15	
	Total	77	15	
T5	GNS	87	16	0.057
	GL	77	15	
	Total	82	16	
T6	GNS	69	20	0.240
	GL	76	16	
	Total	72	18	
T7	GNS	76	22	0.980
	GL	76	16	
	Total	76	19	
T8	GNS	76	15	0.966
	GL	76	15	
	Total	76	15	

T1: initiation of anaesthesia maintenance, T2: attainment of 1.2% FEiso, T3: commencement of the surgical procedure, T4: irrigation of the suspensory ligament of the left ovary, T5: surgical manipulation of the left ovary, T6: irrigation of the suspensory ligament of the right ovary, T7: surgical manipulation of the right ovary, T8: completion of the surgical procedure and discontinuation of anaesthesia. Differences in mean values between groups were considered significant at $p < 0.05$.

Table S2. Descriptive results (mean ± SD) for f_R measurements in 38 dogs undergoing ovariohysterectomy that received a splash block on the suspensory ligaments with 0.5 ml of either lidocaine 2% (GNS) or saline (GL), followed by a gentle one-minute massage. (*n* = 19).

Time Point	Group	Mean	Std. Deviation	p-value
T1	GNS	9	4	0.486
	GL	10	4	
	Total	10	4	
T2	GNS	9	4	0.473
	GL	10	4	
	Total	10	4	

T3	GNS	10	4	0.300
	GL	11	4	
	Total	10	4	
T4	GNS	10	3	0.563
	GL	11	5	
	Total	11	4	
T5	GNS	21	10	0.008*
	GL	13	7	
	Total	17	9	
T6	GNS	10	4	0.077
	GL	13	6	
	Total	11	5	
T7	GNS	15	11	0.289
	GL	12	6	
	Total	14	9	
T8	GNS	13	11	0.321
	GL	11	4	
	Total	12	8	

T1: initiation of anaesthesia maintenance, T2: attainment of 1.2% FEiso, T3: commencement of the surgical procedure, T4: irrigation of the suspensory ligament of the left ovary, T5: surgical manipulation of the left ovary, T6: irrigation of the suspensory ligament of the right ovary, T7: surgical manipulation of the right ovary, T8: completion of the surgical procedure and discontinuation of anaesthesia. *Differences in mean values between groups were considered significant at $p < 0.05$.

Table S3. Descriptive results (mean ± SD) for MAP measurements in 38 dogs undergoing ovariohysterectomy that received splash block on the suspensory ligaments with 0.5 ml of either lidocaine 2% (GL) or saline (GNS), followed by a gentle one-minute massage. (n = 19).

Time point	Group	Mean	Std. Deviation	p-value
T1	GNS	86	12	0.988
	GL	86	10	
	Total	86	11	
T2	GNS	86	9	0.341
	GL	83	12	
	Total	84	10	
T3	GNS	87	9	0.068
	GL	81	11	
	Total	84	10	
T4	GNS	89	11	0.107
	GL	83	9	
	Total	86	10	
T5	GNS	105	13	< 0.001*
	GL	86	9	
	Total	95	15	
T6	GNS	96	9	0.003*
	GL	86	10	
	Total	91	11	
T7	GNS	102	11	< 0.001*
	GL	87	8	

	Total	95	12	
T8	GNS	92	7	0.007*
	GL	84	9	
	Total	88	9	

T1: initiation of anaesthesia maintenance, T2: attainment of 1.2% FEiso, T3: commencement of the surgical procedure, T4: irrigation of the suspensory ligament of the left ovary, T5: surgical manipulation of the left ovary, T6: irrigation of the suspensory ligament of the right ovary, T7: surgical manipulation of the right ovary, T8: completion of the surgical procedure and discontinuation of anaesthesia. *Differences in mean values between groups were considered significant at $p < 0.05$.
